# SF-BARI Score for Assessment of Long-Term Results in Patients with BMI ≥ 50 kg/m^2^ Submitted to Roux-en-Y Gastric Bypass or Sleeve Gastrectomy

**DOI:** 10.1007/s11695-025-07973-8

**Published:** 2025-06-16

**Authors:** André Costa-Pinho, João Araújo-Teixeira, Sara Rodrigues, Hugo Santos-Sousa, Fernando Resende, John Preto, Eduardo Lima-da-Costa

**Affiliations:** 1 Integrated Responsability Center for Obesity (CRI-O), São João Local Health Unit, Porto, Portugal; 2 Faculty of Medicine, University of Porto, Porto, Portugal; 3 General Surgery Department, São João Local Health Unit, Porto, Portugal

**Keywords:** SF-BARI Score, Long-term outcomes, Metabolic and Bariatric Surgery, BMI above 50kg/m2, Roux-en-Y Gastric Bypass, Sleeve Gastrectomy

## Abstract

**Background:**

Patients with a body mass index (BMI) ≥ 50 kg/m^2^ present significant challenges in terms of treatment options, and data on long-term outcomes following metabolic and bariatric surgery (MBS) in this population remain limited. This study aims to explore the long-term outcomes of Roux-en-Y gastric bypass (RYGB) and sleeve gastrectomy (SG) in patients with BMI ≥ 50 kg/m^2^, utilizing the Swiss-Finnish Bariatric Metabolic Outcome Score (SF-BARI Score)—a comprehensive tool that incorporates multiple outcome dimensions—and to identify factors that may influence these outcomes.

**Methods:**

Retrospective cohort study of patients with BMI ≥ 50 kg/m^2^ submitted to RYGB or SG between January 2010 and September 2021, with more than 5 years of follow-up. Several data were collected, and the SF-BARI score was calculated and analyzed. Statistical analysis was performed to identify variables that influenced the score.

**Results:**

We identified 89 patients with long-term follow-up (mean 96.6 months, SD 18.9) after RYGB or SG. The mean SF-BARI score was 94.2 (SD 29.5), with most patients’ outcomes categorized as good. Seventy-six (85.4%) patients had %TWL ≥ 20, but only 34.8% of patients had a final BMI < 35 kg/m^2^. Statistical analysis revealed that younger patients have higher SF-BARI scores related to comorbidities improvement.

**Conclusion:**

Our findings suggest that both RYGB and SG lead to satisfactory long-term outcomes for treatment of patients with BMI ≥ 50 kg/m^2^, according to the SF-BARI score. However, only one-third achieved a BMI < 35 kg/m^2^. Younger patients seem to achieve better results, particularly in comorbidity improvements. MBS outcomes should be reported in a standardized manner, addressing key components such as weight loss, improvement in comorbidities, complications, and quality of life.

## Introduction

Severe obesity is on the rise globally, exerting a profound impact on various diseases, quality of life, and health-related costs [1, 2]. Among patients with obesity, those with a BMI ≥ 50 kg/m^2^ present unique challenges related to increased complications. Nonetheless, this subset of patients may benefit the most from effective treatments.

Metabolic and bariatric surgery (MBS) remains the most successful long-term treatment for obesity and its metabolic complications. In patients with BMI ≥ 50 kg/m^2^, MBS is considered a crucial treatment that may achieve sustainable outcomes, but many challenges arise when considering surgery for this high-risk group of patients [3]. In a large cohort study that included 173,110 patients with BMI ≥ 50 kg/m^2^ submitted to Roux-en-Y gastric bypass (RYGB) or sleeve gastrectomy (SG), it was concluded that BMI ≥ 50 kg/m^2^ remains an independent risk factor for serious complications and the greatest independent risk factor for 30-day postoperative mortality [4].

Furthermore, preoperative BMI has been shown to significantly influence weight loss outcomes after MBS [5]. Patients with higher initial BMI tend to retain a higher final BMI, even when achieving substantial weight loss. Baseline BMI is a critical predictor of weight nadir [6], underscoring the need for cautious goal-setting and clear communication of expected outcomes to surgical candidates with BMI ≥ 50 kg/m^2^.

There is a scarcity of published long-term outcomes for MBS in patients with BMI ≥ 50 kg/m^2^, as highlighted by recent systematic reviews and meta-analyses [7, 8]. Moreover, few studies extend beyond 5 years of follow-up. Standardizing outcome reporting after MBS is crucial to ensure consistency in this field [9]. Historically, outcomes following MBS have been primarily evaluated on weight loss [10–12], but in recent years, there has been a growing emphasis on multidimensional parameters, including the resolution of associated comorbidities, enhancement of health-related quality of life, and the incidence of postoperative complications. The Swiss-Finnish Bariatric Metabolic Outcome Score (SF-BARI Score) is a freely available, web-based instrument that integrates these critical domains into a single comprehensive measure. Although this score has not yet been specifically validated in patients with a BMI > 50 kg/m^2^, we elected to explore its utility as a holistic tool for assessing postoperative outcomes in this high-risk population.

Our study aims to explore the long-term results of RYGB and SG in patients with BMI ≥ 50 kg/m^2^, using the SF-BARI score. As a secondary objective, we assessed the variables that may influence long-term outcomes after MBS in patients with BMI ≥ 50 kg/m^2^.

## Materials and Methods

Data access was granted after approval by the Ethical Committee and Data Protection Officer.

### Study Population

We performed a retrospective cohort study of all adult patients (18 to 65 years) submitted to MBS between January 2010 and September 2021 in a single center. We selected patients with BMI ≥ 50 kg/m^2^, submitted to RYGB or SG, with follow-up ≥ 5 years after MBS. Patients submitted to RYGB or SG after adjustable gastric band were excluded. Patients submitted to secondary revisional surgeries after initial RYGB or SG were included only if last follow-up ≥ 5 years was available after last surgery.

### Preoperative Protocol

All patients were submitted to a rigorous preoperative protocol, including extensive blood and urine analysis, upper endoscopy, abdominal sonography and barium swallow abdominal radiographs, and were offered a multidisciplinary approach including pre- and post-operative consultations with a bariatric surgeon, endocrinologist, psychiatrist, and a dietitian. Most patients were also evaluated preoperatively by anesthesiology, pneumology, and cardiology to further stratify risk and/or optimize comorbidities.

### Surgical Treatment

Surgical techniques were standardized and performed by a team of five surgeons. In RYGB, we used a small gastric pouch of approximately 4 × 8 cm, the gastrojejunostomy was calibrated with a 36Fr bougie, and performed side-to-side using a linear stapler. The limbs lengths were 100 cm for the biliopancreatic limb and 120 cm for the alimentary/Roux limb. In SG, we preserved 6 cm of antrum and used a 48Fr bougie for calibration. Between 2009 and 2016, all patients with predictable high risk for surgery (e.g., central obesity, chronic respiratory disease) had intra-gastric balloon (IGB) for 6 months before proceeding to surgery.

### Follow-up

As per the selection criteria, all patients had a minimum follow-up period of at least 5 years after the last MBS. The follow-up included annual consultations with a surgeon, endocrinologist, dietitian, and psychologist. All patients submitted to MBS in our institution are advised to take high-dose multivitamin supplementation. Additionally, annual blood and urine analyses were performed.

### Clinical Data Evaluated

Several variables were examined including demographic characteristics, preoperative BMI, use of IGB before surgery, surgical procedure performed, postoperative surgical complications assessed using the comprehensive complication index, weight loss outcomes (percentage total weight loss %TWL, percentage excess weight loss %EWL, final BMI), and major comorbidities before and after surgery. The need for revisional surgery was also evaluated.

The SF-BARI score [13] was employed: SF-BARI Score Calculator 1.0 available at https://sites.utu.fi/sfbariscore/calculator. This score encompasses %TWL, improvement of comorbidities (diabetes, dyslipidemia, hypertension, and obstructive sleep apnea), and the comprehensive complication index. The SF-BARI score generates a continuous variable ranging from − 100 to + 200 that can be grouped into five distinct categories: suboptimal, fair, good, very good, and excellent. Assessment of quality of life was not performed in this study.

### Statistical Analysis

The data were analyzed using IBM SPSS Statistics 29.0® (SPSS Inc., Chicago, IL, USA). Statistical significance (*p*-value) was assumed for 5%. In descriptive statistics, central values were described as mean or median for variables with normal or non-normal distribution, respectively. The preferred dispersion measures were standard deviation (SD) for mean, and interquartile range (IQR) for median. Variables were assessed for normality using both visual (histogram and Q-Q plot) and Shapiro–Wilk test.

Assessment of variables influencing SF-BARI score was performed. Standard parametric tests (*T*-student) were used for comparison of variables with normal distributions (after Levene test for variance). Relations between continuous variables were studied using the Spearman correlation test.

No sample size calculation was performed, which limits the statistical interpretation.

## Results

Between January 2010 and September 2021, MBS was performed in 4014 cases, including 172 duplicates (revisional procedures for the same patients), in a total of 3842 different patients. RYGB was performed in 2529 cases (63.0%). Mean BMI was 43.3 kg/m^2^ (SD 5.8), and 469 (11.7%) patients had a BMI ≥ 50 kg/m^2^. Long-term (> 5 years) follow-up was available for 19.0% of patients with BMI ≥ 50 kg/m^2^.

Applying the selection criteria described, we had a total of 89 patients with BMI ≥ 50 kg/m^2^ submitted to RYGB or SG, with the last available follow-up > 5 years since the last MBS. Most patients were female (80.9%), the median initial BMI was 53.0 kg/m^2^ (IQR 4.3), and the most performed surgery was RYGB (66.3%). Demographic and perioperative data are presented in Table 1.

**Table 1:** Demographics and perioperative data (*n* = 89)

Age (years)	Mean (SD)	44.0	(11.7)
Female gender	*n* (%)	72	(80.9)
Initial BMI (kg/m^2^)	Median (IQR)	53.0	(4.3)
Preoperative BMI (kg/m^2^)	Median (IQR)	52.4	(4.0)
IGB before surgery	*n* (%)	19	(21.3)
Diabetes	*n* (%)	37	(41.6)
Dyslipidemia	*n* (%)	28	(31.5)
Hypertension	*n* (%)	57	(64.0)
Surgery performed	*n* (%)	RYGB = 59 SG = 30	(66.3) (33.7)

*SD*, standard deviation; *IQR*, interquartile range (Q1–Q3); *IGB*, intragastric balloon; *RYGB*, Roux-en-Y gastric bypass; *SG*, sleeve gastrectomy

In this series, 19 (21.3%) patients had an intra-gastric balloon previously to surgery that resulted in a median %TWL of 8.3% (minimum 6.9%; maximum 32.4%; IQR = 12.3). There were three patients who required secondary surgery for insufficient weight loss. Those 3 patients were initially submitted to sleeve gastrectomy and, 22, 25, and 38 months later, were converted to RYGB. Note that many other patients with initial BMI ≥ 50 kg/m^2^ were submitted to secondary procedures but not included in this series because they did not meet the selection criteria of last available follow-up above 5 years since the last MBS. All surgeries were performed by laparoscopic approach, without any conversion to laparotomy.

Several outcomes are presented in Table 2, including the SF-BARI score and its components: complications, weight loss, and comorbidity outcomes.

**Table 2:** Outcomes at last available follow-up (*n* = 89)

Follow up (months)	Mean (SD)	96.6	(18.9)
SF-BARI score	Mean (SD)	94.2	(29.5)
SF-BARI score—categories			
Suboptimal (< 35)	*n* (%)	2	(2.2)
Fair (35 to < 70)	*n* (%)	15	(16.9)
Good (70 to < 110)	*n* (%)	44	(49.4)
Very good (110 to < 135)	*n* (%)	23	(25.8)
Excellent (≥ 135)	*n* (%)	5	(5.6)
Complications			
Major surgical complications	*n* (%)	1	(1.1)
CCI	Median (min–max)	0	(0–20.9)
Mortality	*n* (%)	0	0
Weight loss			
% TWL	Median (IQR)	31.8	(13.3)
Patients with %TWL > 20	*n* (%)	76	(85.4)
% EWL	Median (IQR)	60.5	(23.3)
Patients with %EWL > 50	*n* (%)	64	(71.9)
Weight lost (kg)	Mean (SD)	44.2	(16.2)
Final BMI (kg/m^2^)	Mean (SD)	37.4	(5.3)
Patients with final BMI < 30 kg/m^2^	*n* (%)	3	(3.4)
Patients with final BMI < 35 kg/m^2^	*n* (%)	31	(34.8)
Patients with final BMI < 40 kg/m^2^	*n* (%)	64	(71.9)
Comorbidities at last follow-up			
Diabetes	*n* (%)	16	(18.0)
Dyslipidemia	*n* (%)	25	(28.1)
Hypertension	*n* (%)	37	(41.6)
Obstructive sleep apnea	*n* (%)	4	(4.5)
Comorbidities improvement*	Mean (SD)	37.9	(16.3)

*SD*, standard deviation; *IQR*, interquartile range (Q1–Q3); *CCI*, comprehensive complication index. * Comorbidity improvement was defined as a part of the SF-BARI score: for diabetes, dyslipidemia, and hypertension, no disease/medication at baseline or at follow-up receives + 10points. Worsened or “de novo” (medication increase/started) − 10 points. Medication unchanged (if disease at baseline) 0 points. Improved receives + 10 points, and remission + 20 points. For obstructive sleep apnea, no CPAP receives + 10 points, and CPAP 0 points. As a result, comorbidity improvement can vary between – 30 points and + 70 points

The mean follow-up was 96.6 months (SD 18.9).

The SF-BARI score had a mean value of 94.2 (SD 29.5). SF-BARI score was categorized in groups: 2.2% of patients had suboptimal scores (< 35), 16.9% had fair scores (35 to < 70), 49.4% had good scores (70 to < 110), 25.8% had very good scores (110 to < 135), and 5.6% had excellent scores (≥ 135).

Regarding complications, the median comprehensive complication index was 0, ranging from 0 to 20.9. There were no mortalities reported, and we only had one major surgical complication during this long period of follow-up: one case of marginal ulcer after RYGB that resulted in upper gastrointestinal bleeding. The patient was readmitted to the hospital several months after surgery. Endoscopic treatment was necessary to control the bleeding. Conservative treatment of the ulcer was posteriorly used, and the patient did not require any other treatments.

Nutritional complications were not considered in this study.

In terms of weight loss at the last follow-up available, the median %TWL was 31.8 (IQR 13.3). A significant majority of patients (85.4%) achieved a %TWL greater than 20. At the final follow-up, the mean BMI was 37.4 kg/m^2^ (SD 5.3), and 34.8% of patients had a final BMI below 35 kg/m^2^.

When examining comorbidities at the last follow-up, 18.0% of patients had diabetes, 28.1% had dyslipidemia, 41.6% had hypertension, and 4.5% had obstructive sleep apnea. The mean improvement in comorbidities was substantial (37.9).

The type of surgery (RYGB vs. SG) did not significantly impact the SF-BARI score or its components. According to the type of surgery performed, the baseline demographics of patients who underwent RYGB were as follows: 52 (88.1%) were female, with a mean age of 42.2 years (SD 10.6), a median pre-operative BMI of 52.8 kg/m^2^ (IQR 4.3), 23 (39.0%) had diabetes, 37 (62.7%) had hypertension, and 10 (16.9%) had dyslipidemia. The baseline characteristics of patients who underwent SG were: 20 (66.7%) were female, with a mean age of 47.7 years (SD 13.0), a median pre-operative BMI of 53.0 kg/m^2^ (IQR 4.8), 14 (46.7%) had diabetes, 20 (66.7%) had hypertension, and 15 (50.0%) had dyslipidemia.

The distribution of patients regarding SF-BARI score (category/score) according to the type of surgery performed and the initial BMI are represented in Graphic 1 and Fig. 1, respectively.

**Graphic 1:**
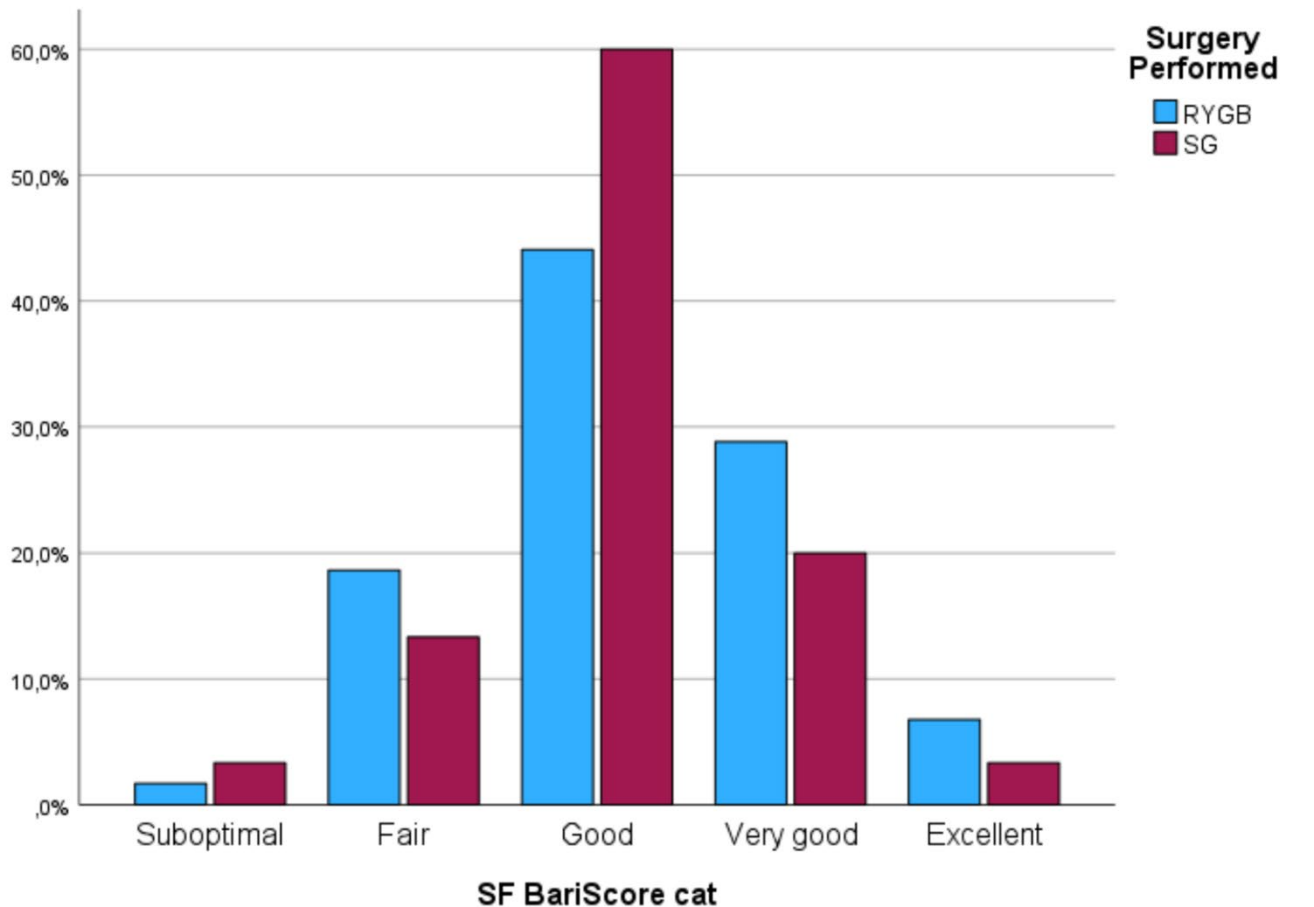
Categories of SF-BARI Score according to surgery performed, at last follow-up

**Fig. 2:**
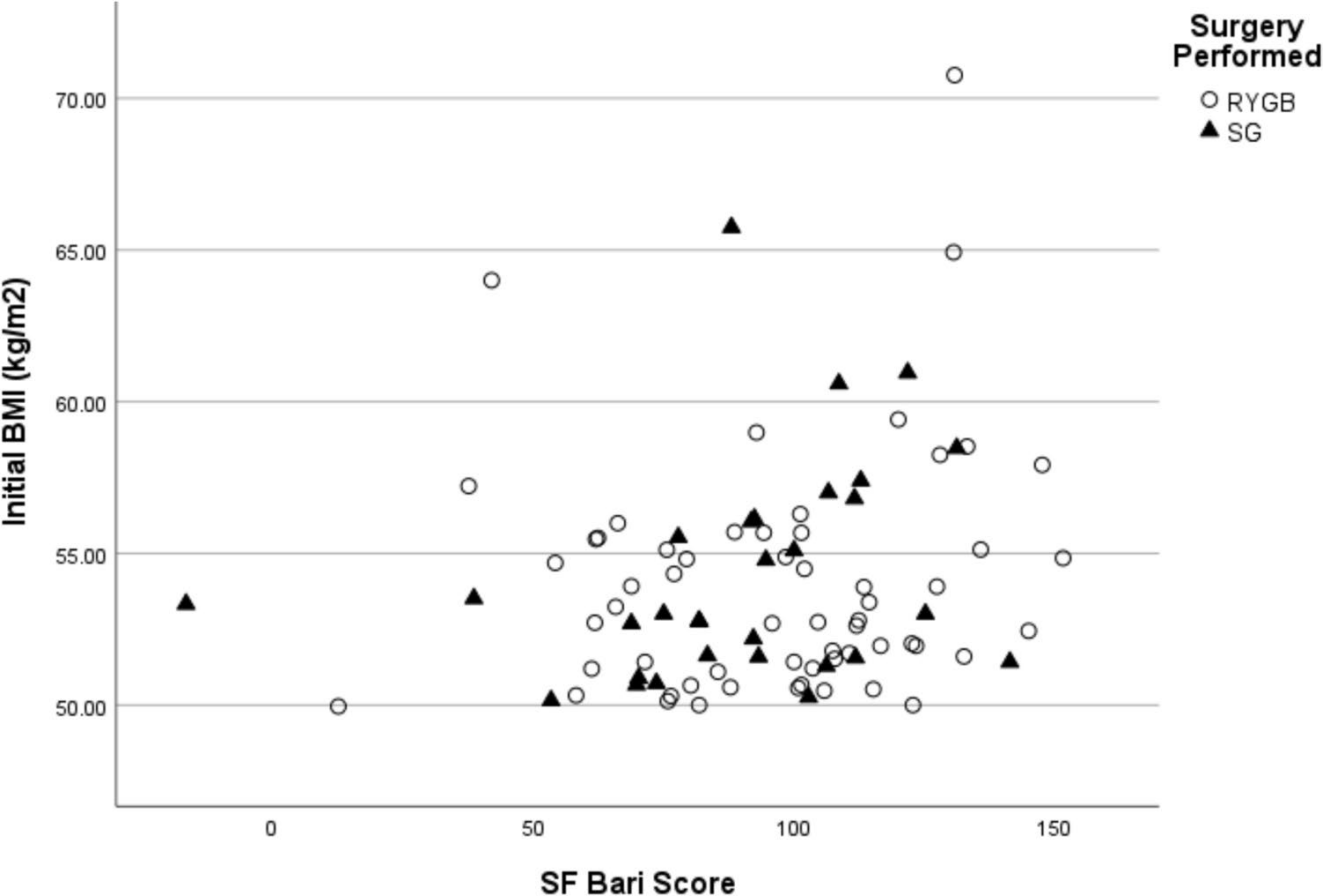
Distribution of patients regarding the relation between SF-BARI Score at last follow-up and the initial BMI, according to surgery performed

Statistical analysis regarding relations between SF-BARI score and type of surgery, gender, age, initial BMI, and diabetes are presented in Table 3.

**Table 3:** SF-BARI score, %TWL, and improvement in comorbidities according to type of surgery, gender, BMI, diabetes, and age

	SF-BARI score Mean (SD)	*P* value	%TWL Mean (SD)		*P *value	Comorbidities improv Mean **(SD)**	*P* value
Type of surgery							
RYGB (***n*** = 59)	96.5 (29.1)	*P* = 0.30	31.0 (9.2)	*P* = 0.24		39.3 (17.3)	*P* = 0.86
SG (***n*** = 30)	89.7 (30.3)		30.5 (10.6)			35.0 (14.1)	
Gender							
Female (***n*** = 72)	96.0 (28.8)	*P* = 0.28	31.0 (9.8)	*P* = 0.62		39.0 (15.5)	*P* = 0.24
Male (***n*** = 17)	86.6 (32.2)		29.8 (9.1)			32.9 (19.3)	
BMI							
BMI > 60 kg/m2 (***n*** = 6)	103.6 (34.2)	*P* = 0.42	37.2 (5.8)	*P* = 0.09		35.0 (10.5)	*P* = 0.66
BMI 50–60 kg/m2 (***n*** = 83)	93.5 (29.3)		30.3 (9.7)			38.1 (16.7)	
Diabetes							
Diabetic (***n*** = 37)	90.0 (37.1)	*P* = 0.30	35.9 (20.6)	*P* = 0.39		28.6 (11.9)	*P* = 0.10
Non-diabetic (***n*** = 52)	97.2 (22.7)		39.2 (12.5)			32.3 (7.4)	
Age							
< 40 years (***n*** = 32)	101.7 (22.9)	*P* = 0.07	32.1 (9.9)	*P* = 0.32		43.1 (6.9)	*P***= **0.005
≥ 40 years (***n*** = 57)	90.0 (32.1)		30.0 (9.5)			34.9 (19.2)	

*%TWL*, percentage of total weight lost; *RYGB*, Roux-en-Y gastric bypass; *SG*, sleeve gastrectomy; *SD*, standard deviation; *BMI*, body mass index

We observed that younger age was statistically associated with higher scores in comorbidities improvement. To assess the strength of this association, we used the Spearman correlation coefficient to determine the rank correlation between the continuous variables (initial BMI and age) and SF-BARI score, %TWL, and comorbidities improvement—Table 4. The analysis revealed that younger patients tend to have higher SF-BARI scores and comorbidities improvement, although with a weak correlation (ρ =  − 0.23 and ρ = − 0.33, respectively).

**Table 4:** Spearman correlation test between initial BMI, age and the outcomes

	SF-BARI score		%TWL		Comorbidities improvement	
	SCC (ρ)	*P* value	SCC (ρ)	*P* value	SCC (ρ)	*P* value
BMI	-	*P* = 0.072	-	*P* = 0.135	-	*P* = 0.413
Age	− 0.23	*P*** = **0.028	-	*P* = 0.242	− 0.33	*P* **< **0.001

*SCC*, Spearman correlation coefficient; *%TWL*, percentage of total weight lost; *BMI*, body mass index

The other variables studied did not seem to statistically influence the SF-BARI score.

## Discussion

We report long-term results of 89 patients with initial BMI ≥ 50 kg/m^2^ submitted to RYGB or SG. The mean follow-up was above 8 years, and all patients had at least 5 years of follow-up after the last MBS.

The outcomes were reported using a comprehensive and standardized score. The SF-BARI score [13] was developed from the merged data from two large randomized clinical trials [14, 15]. This score considers different outcomes after surgery: weight loss, comorbidities improvement, complications, and optionally, quality of life. The easy-to-use scoring system, based on a simple website, provides a continuous and categorical variable that sums up all the relevant information regarding outcomes after MBS. It is important to emphasize that the SF-BARI score has not been specifically validated for patients with a BMI ≥ 50 kg/m^2^. While the studies supporting this scoring system did not exclude these patients, we must stress that only a limited number of patients with a BMI ≥ 50 kg/m^2^ were included [14, 15]. Therefore, we were particularly interested in evaluating how the SF-BARI score would reflect outcomes in this subset of patients.

In our study, at the last follow-up available, 81% of patients achieved good, very good, or excellent results according to the SF-BARI score. This is in line with 85% of patients reaching %TWL > 20, and 72% of patients %EWL > 50, which are commonly referred to as markers of adequate weight loss after MBS. As expected, we also observed a high rate of comorbidities improvement and a low complication rate.

Although most patients achieved a good or better category in the long term, fewer than 35% attained a BMI < 35 kg/m^2^, and less than 3.5% were classified as non-obese at long term after MBS. As previously stated, pre-operative BMI is a critical predictor of weight nadir after MBS [6, 16, 17]. As a result, and consistent with other authors [12], a BMI < 40 kg/m^2^ may be considered a satisfactory weight loss outcome at long-term follow-up. In our series, 72% of patients achieved a final BMI < 40 kg/m^2^, aligning more closely with the outcomes obtained by the SF-BARI score.

Patients with BMI ≥ 50 kg/m^2^ should be thoroughly informed about the potential challenges in achieving an optimal weight even after undergoing MBS. It is also important to emphasize that this limitation may restrict access to body contouring surgeries, as most plastic surgeons set a BMI threshold of 28–30 kg/m^2^ for such procedures due to the increased risk of wound healing complications [18]. Comprehensive counselling, careful management of expectations, and clear communication regarding objectives, risks, and benefits of MBS are essential for all patients, particularly in this subset of patients. It is also important to raise awareness among plastic surgeons regarding the need to thoroughly investigate the actual complication rates in patients with a high BMI following MBS, as the body composition and inflammatory status of these patients may differ from those of individuals with a similar BMI who have not undergone MBS.

Recent systematic reviews and meta-analyses [7, 8] comparing long-term outcomes of RYGB and SG in patients with BMI ≥ 50 kg/m^2^, suggested that RYGB leads to improved outcomes at 12 months, but no differences were found at 24 or 36 months. There is conflicting published data regarding which surgery may be the best for patients with BMI ≥ 50 kg/m^2^. In our study, the type of surgery (RYGB or SG) did not significantly impact the SF-BARI Score or its components. Nonetheless, we observed that RYGB consistently demonstrated higher mean weight loss, greater resolution of comorbidities, higher percentage of patients achieving a final BMI < 35 kg/m^2^, and higher mean SF-BARI score. These differences, however, did not achieve statistical significance, which may be explained by the small population sample.

As for surgical complications, some studies report that RYGB is related to higher rates of reoperation and readmission at 30 and 90 days [19], but this data was inconsistent with our findings and other studies [20, 21]. It seems that both surgeries have a high safety profile even in patients with BMI ≥ 70 kg/m^2^ when performed by experienced teams [22].

Some patients with BMI ≥ 50 kg/m^2^ may require more effective strategies to achieve improved outcomes, which means considering enhanced metabolic surgeries and/or two-step approaches combining pharmacotherapy or endoscopic therapy with subsequent MBS. Some studies suggest that surgeries such as one-anastomosis gastric bypass (OAGB) [23], single anastomosis duodenal-ileostomy with sleeve (SADI-S) [24], or biliopancreatic diversion with duodenal switch (BPD-DS) [25] may be more effective for patients with BMI ≥ 50 kg/m^2^, although probably with higher nutritional complications.

We examined the statistical association of SF-BARI score with other variables such as age, gender, diabetes, and initial BMI, but significant associations were only found with age: younger patients (18–39 years) have higher SF-BARI scores in the long term after bariatric surgery, particularly regarding improvement in comorbidity scores. The analysis of continuous variables confirmed these findings, but the correlation strength was low. It must be noted that the higher scores observed in the young population were mostly explained by the improvement of comorbidities rather than %TWL or surgical complications. This greater improvement in comorbidities observed among younger patients may be attributed to earlier stages of disease, lower baseline burden, better adherence to medication, or a more favourable impact on metabolic and hormonal profiles.

The SF-BARI score has limitations that may impact its clinical application. A key issue is the potential overlap of outcomes; for instance, a resolved complication like Petersen’s hernia might still significantly affect the score despite minimal long-term impact. Additionally, the score does not address nutritional complications, which are common after MBS and crucial for patient health [26]. It also omits consideration of obesity improvement goals, such as using MBS as a bridge to other surgeries (e.g., orthopedic or transplantation), limiting its ability to reflect progress toward critical outcomes. Furthermore, the SF-BARI score focuses on a narrow set of comorbidities—diabetes, dyslipidemia, hypertension, and obstructive sleep apnea—excluding other conditions that influence surgical success and long-term health. Despite these limitations, the SF-BARI score offers a simple and intuitive tool for evaluating outcomes after MBS. In patients with a BMI > 50 kg/m^2^, in particular, we consider that the SF-BARI score satisfactorily captures the complexity of outcomes by incorporating %TWL, encompassing a broader spectrum of outcome measures beyond weight loss alone, and stratifying results into clinically meaningful categories.

We must acknowledge some limitations in our study. We had a small number of patients, especially those with a BMI > 60 kg/m^2^ (only 6 patients, 6.7%). These patients with very high BMI are often lost to follow-up, which could be attributed to various factors such as unsatisfactory outcomes leading to loss of motivation, greater psychosocial sequelae [27], or mobility difficulties. Thus, the selection criteria requiring available follow-up data above five years likely selected the most motivated patients who continued in follow-up, and probably those with more satisfactory results. A comparison of the baseline characteristics of the 89 patients with long-term follow-up and the remaining 380 patients with a BMI > 50 kg/m^2^ but a follow-up < 5 years could have been informative in addressing that potential risk of bias.

Another potential source of bias is that we did not analyse the use of non-surgical treatments that patients might have undergone during the follow-up period. There is also some admitted heterogeneity in our population, as some patients underwent intragastric balloon placement before surgery. We did not integrate the quality of life in the score, so no conclusions could be made about this very relevant topic. The statistical analysis of our results should be interpreted with caution, as the study involved a small number of patients, and no sample size calculation was performed. Under these conditions, both significant and non-significant P-values have limited interpretative value.

## Conclusion

Most patients with a BMI ≥ 50 kg/m^2^ who underwent RYGB or SG achieved good or better outcomes at long-term follow-up, as measured by the SF-BARI score. These findings indicate that both procedures provide satisfactory long-term results considering weight loss, comorbidity improvement, and safety. However, only one-third of patients achieved a BMI < 35 kg/m^2^ at long-term follow-up, emphasizing the potential need for more ambitious multimodal treatment approaches.

The SF-BARI score is a valuable and intuitive tool for standardizing and comparing MBS outcomes, with potential for clinical application, though further refinements are warranted.

## Data Availability

No datasets were generated or analysed during the current study.
